# Aurora-A overexpression enhances cell-aggregation of Ha-*ras *transformants through the MEK/ERK signaling pathway

**DOI:** 10.1186/1471-2407-9-435

**Published:** 2009-12-12

**Authors:** Ya-Shih Tseng, Jenq-Chang Lee, Chi-Ying F Huang, Hsiao-Sheng Liu

**Affiliations:** 1 Department of Medical Technology, Chung Hwa University of Medical technology, Tainan, Taiwan; 2 Department of Surgery, College of Medicine, National Cheng Kung University Hospital, Tainan, Taiwan; 3 Institute of Clinical Medicine, National Yang-Ming University, Taipei, Taiwan; 4 Department of Microbiology and Immunology, College of Medicine, National Cheng Kung University, Tainan, Taiwan; 5 Center for Gene Regulation and Signal Transduction Research, National Cheng Kung University, Tainan, Taiwan

## Abstract

**Background:**

Overexpression of Aurora-A and mutant Ras (Ras^V12^) together has been detected in human bladder cancer tissue. However, it is not clear whether this phenomenon is a general event or not. Although crosstalk between Aurora-A and Ras signaling pathways has been reported, the role of these two genes acting together in tumorigenesis remains unclear.

**Methods:**

Real-time PCR and sequence analysis were utilized to identify Ha- and Ki-*ras *mutation (Gly -> Val). Immunohistochemistry staining was used to measure the level of Aurora-A expression in bladder and colon cancer specimens. To reveal the effect of overexpression of the above two genes on cellular responses, mouse NIH3T3 fibroblast derived cell lines over-expressing either Ras^V12^and wild-type Aurora-A (designated WT) or Ras^V12 ^and kinase-inactivated Aurora-A (KD) were established. MTT and focus formation assays were conducted to measure proliferation rate and focus formation capability of the cells. Small interfering RNA, pharmacological inhibitors and dominant negative genes were used to dissect the signaling pathways involved.

**Results:**

Overexpression of wild-type Aurora-A and mutation of Ras^V12 ^were detected in human bladder and colon cancer tissues. Wild-type Aurora-A induces focus formation and aggregation of the Ras^V12 ^transformants. Aurora-A activates Ral A and the phosphorylation of AKT as well as enhances the phosphorylation of MEK, ERK of WT cells. Finally, the Ras/MEK/ERK signaling pathway is responsible for Aurora-A induced aggregation of the Ras^V12 ^transformants.

**Conclusion:**

Wild-type-Aurora-A enhances focus formation and aggregation of the Ras^V12 ^transformants and the latter occurs through modulating the Ras/MEK/ERK signaling pathway.

## Background

The role of Aurora-A, a serine/threonine kinase, in tumorigenesis has been reported [1-4]. In proliferative cells, the expression levels of Aurora-A mRNA and protein are low during G1 and S phases. The levels peak at G2 phase and fall during mitotic exit and G1 phase of the next cell cycle [3,5]. Aurora-A protein consists of 403 amino acids and has a molecular weight of 46 kilo Daltons (kDa) [5]. Overexpression of Aurora-A has been detected in several human cancer cell lines and cancers of the following tissues: bladder, breast, colon, liver, gingival, gliomas, medulloblastoma, ovarian, pancreas, prostate and tongue [6-16]. Ectopic expression of Aurora-A in mouse NIH3T3 cells and Rat1 fibroblasts causes centrosome amplification and cell transformation [8,17]. This suggests that Aurora-A gene amplification and overexpression play a role in human carcinogenesis, largely due to the effect of Aurora-A on oncogenic cell growth, rather than a loss of maintenance of centrosomal or chromosomal integrity.

Ras proteins are important for controlling the activities of several crucial signaling pathways. The *ras-*gene encoded proteins become constitutively active due to point mutations in their coding sequences, especially at amino acid 12, 13, and 61 [18]. These activated Ras proteins contribute significantly to several aspects of the malignant phenotype, including deregulation of tumor-cell growth, programmed cell death, invasiveness, and induction of new blood-vessel formation [19].

Various Ras-regulated signaling pathways are responsible for cell survival, transformation, and apoptosis [20,21]. Multiple effectors have been found downstream of Ras, including Raf, PI3K, RalGDS, RIN1, MEKK, GAP, NF1, and AF6 [21]. Overexpression of Ha-*ras*^val12 ^oncogene not only transforms NIH3T3 cells but also sensitizes them to various stresses, such as serum depletion, Lovastatin, tumor necrosis factor-α and 5-FU treatments [22-26]. Through the Ras/Raf interaction, Raf activates MEK1/2, which subsequently phosphorylates ERK1/2 and activates the transcription factor, Elk [27,28]. After activation, Elk complexes with the serum responsive factor (SRF) and binds to the serum responsive element (SRE) which is an important element in the *c-fos *promoter [29-31]. RalGDS, another Ras effector, associates with Ras and activates Ral (a small GTPase), including RalA and RalB [32].

Studies on progesterone-induced maturation of *Xenopus *oocytes indicate that overexpression of kinase Eg2, a *Xenopus *member of the Aurora/Ipl1 family, activates the MAP kinase pathway [33]. This study raises the possibility that Aurora protein may also transduce cell transformation signals through the MAPK signaling pathway. In addition, Aurora-A could associate with NM23-H1, which may phosphorylates the scaffold kinase repressor of Ras (KSR) [34-36]. Gigoux et al., (2002) reported that the interaction between Aurora-A and RasGAP, a negative Ras regulator, decreased the kinase activity of Aurora-A [37]. Wu et al., (2005) found that RalGDS and RalA are downstream substrates of Aurora-A [38]. Tatsuka et al., (2005) showed that overexpression of Aurora-A potentiated Ha-*ras*-mediated oncogenic transformation by increasing focus formation [39]. Furukawa et al., (2006) showed that Aurora-A is one of the downstream targets of MAPK signaling [40]. These observations imply some degree of crosstalk between Aurora-A and Ras signaling pathways.

In this study, the collective role of Aurora-A and Ha-*ras *in cell aggregation was unraveled. The possible signaling pathways involved were also investigated.

## Methods

### Tumor Tissues

The cancer tissues from National Cheng Kung University Hospital between 2001 and 2004 were eligible for analysis. Consent from the patients was obtained, and the study was approved by the institutional review board.

### Genomic DNA preparation

The tissues were homogenized with a mortar and a pestle in the presence of liquid nitrogen, followed by phenol/chloroform extraction. After ethanol precipitation, genomic DNA was dissolved in TE buffer.

### Detection of Ha- and Ki-ras codon 12 mutation

Detection of Ha-*ras *codon 12 mutation was conducted using a commercial SNP system (ABI, USA) [41]. Detection of Ki-*ras *codon 12 mutation was conducted using a commercial SNP system following the manufacturer's instructions [42] (Roche, Germany).

### Plasmids

The wild-type and catalytic-inactive mutant Aurora-A genes were cloned into pEGFPN1 plasmid (pEGFP-Aurora-A-WT and pEGFP-Aurora-A-KD). The construction of pHARalAS183A and pHARalS194A was described previously [38].

### Cell lines and culture

The NIH3T3 cell harbors the inducible Ha-*ras*^V12 ^oncogene (pSVlacO *ras*) designated as 7-4 [22]. The stable cell lines Vector, WT and KD were derivatives of 7-4 cells containing GFP (pEGFPN1), wild-type GFP-Aurora-A (pEGFP-Aurora-A- WT) as well as kinase-inactivated GFP-Aurora-A (pEGFP-Aurora-A-KD), respectively. All the fibroblast stable cell lines were maintained in Dulbecco's modified Eagle medium (DMEM; GIBCO, USA) supplemented with 10% calf serum (GIBCO) at 37°C in a 5% CO_2 _incubator.

### Immunohistochemical (IHC) staining

Tissue sections of paraffin embedded specimens on the slides after deparaffinization and rehydration. Then, the slides were soaked in 1× PBS for 5 min and immersed in 1.6% H_2_O_2 _(in methanol) for 5 min at room temperature (RT). After rinsing with 1× PBS, the slides were incubated with boiling citric acid (10 mM) twice for 5 min and the slides were rinsed with 1× PBS. Then, the specimens were incubated with primary antibody at 4°C for overnight. On the second day, the slides were rinsed 3 times for 5 min with 1XPBS. Then, the slides were incubated with biotinylated secondary antibody (DakoCytomation, LSAB2 System-HRP, USA) for 10 min at RT. After rinsing the slides 3 times for 5 min with 1× PBS Streptavidin reagent (DakoCytomation) was applied to cover the specimens for 10 min at RT. The slides were rinsed again 3 times for 5 min with 1× PBS. AEC solution (DakoCytomation) was added to cover specimens for 10 min at RT. The specimens were rinsed gently with distilled water and counter stained with 10% hematoxylin. Finally, the slides were rinsed gently with distilled water and mounted.

### Establishment of stable cell lines

After seeding cells on the culture plate for overnight, the medium was replaced with fresh medium. The desired plasmid DNA precipitated with ethanol was resuspended with 40 μl of sterile H_2_O. Then, 0.5 ml of CaCl_2 _(pH 7.9) solution was mixed with the DNA solution, transferred into a 3 ml tube and mixed with 0.5 ml of HEPES buffer (pH 7.1). The calcium-DNA solution was transferred into the cell culture plate and the cells were further incubated at 37°C in a humidified incubator with 5% CO_2_. Six hours after incubation, the medium was replaced with medium containing serum and incubated for another 24 hr. The cells were then treated with the antibiotic G418 (Sigma, USA) to select for drug-resistant cell lines. Within 10 to 14 days, the cells containing the antibiotic resistance gene formed colonies, which were selected, propagated and analyzed for transgene expression by Western blotting.

### Cell growth assay

Cell growth was determined by MTT assay. The cells (1 × 103/well) were plated in 96-well plates. After incubation with or without IPTG (2.5 mM) for the indicated times, the cells were treated with 10 μl of MTT solution (5 mg/ml, Sigma, USA) and incubated for another 3 h at 37°C. Finally, 100 μl DMSO were added to lyses the cells, the absorbance of the cell lysates was measured at 540 nm by a Dynatech Mr 5000 microplate reader (Dynatech laboratories, USA).

### Focus formation assay

The cells (5 × 10^2^) were plated on 10 cm plates with or without IPTG (2.5 mM). Media with or without IPTG were changed every 3-4 days for 2 weeks. The cells were washed twice, and then fixed with 4% paraformaldehyde for 10 min at 37°C. The paraformaldehyde was then aspirated from the plates, and washed twice with 1× PBS. Giemsa solution (Sigma, USA) was added to cover the bottom of the plate. After incubation at RT for 5 min, Giemsa solution was poured off, and the plates were rinsed in double distilled H_2_0 until excess color ceased coming off. The plates were dried at RT and the foci were counted.

### RalA pull-down assay

The cells were lysed in lysis buffer (50 mM Tris-HCl, pH 7.4, 5 mM MgCl_2_, 1% Nonidet P-40, 150 mM NaCl, 1 mM phenylmethylsulfonyl fluoride). Total cell lysates (500 μg) were incubated for 1 h at 4°C with 50 μl of glutathione beads (Sigma, USA) coated with GST-RalBD that had been produced in *Escherichia coli*. Then, the beads were washed three times with lysis buffer and boiled in the sample buffer. Samples were resolved on a 12% SDS-PAGE, followed by Western blot analysis using anti-RalA antibody [43,44].

### Western blot analysis

Cell lysates (50 μg) were subjected to 12% SDS-PAGE and subsequently transferred to a PVDF membrane (Millipore, USA). The membranes were blocked with 5% non-fat milk for 1 h at RT. The membranes were washed with anti-Aurora-A (Transduction, BD, Germany), anti-AKT (Cell signaling technology), anti-p-AKT(Thr308) (Cell signaling technology), anti-Ras (Oncogene, USA), anti-p-MEK(Ser217/221) (Cell Signaling technology, USA), anti-ERK1/2 (Cell Signaling technology), anti-p-ERK1/2 (Thr202/Tyr204) (Cell signaling technology), anti-p-H3S10 (Cell signaling technology), and anti-β-actin (Sigma) antibodies. The reaction was followed by probing with peroxidase-coupled secondary antibodies and then detected by enhanced chemiluminescence (Amersham Pharmacia, USA).

### Statistical Analysis

Densitometry data were represented as fold increase. Student's *t *test was used to analyze the comparisons of differences, and p = 0.01 was considered significant.

## Results

### Detection of Aurora-A overexpression accompanied with Ha-*ras *mutation in bladder cancers

**Figure 1:**
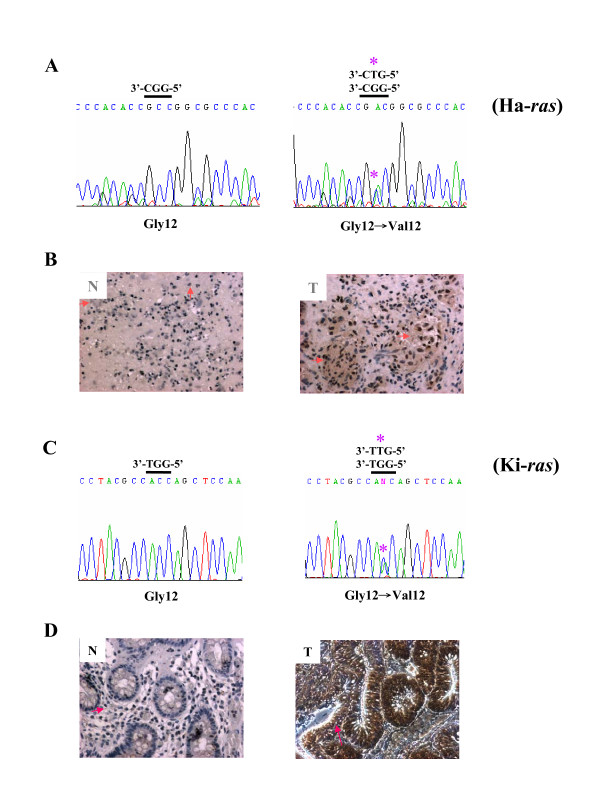
**Detection of Ha- and Ki-RasG12V mutation and overexpression of Aurora-A in bladder and colon cancer specimens**. (A) Sequence analysisof Ha-*ras *codon 12 mutations of bladder cancer specimen shows the Gly12 to Val12 mutation of Ha-*ras *gene. (B) Aurora-A protein expression in bladder cancer (T) and normal tissue (N) was detected by IHC staining using anti-Aurora-A specific antibody. (C) Sequence analysisof Ki-*ras *codon 12 mutations of colon cancer specimen shows the Gly12 to Val12 mutation of Ki-*ras *gene. (D) Aurora-A protein expression in colon cancer (T) and normal tissue (N). " * " indicates the mutation site of *ras *gene. The pink arrow points the expression of Aurora-A by IHC stain.

Aurora-A overexpression accompanied with Ha-*ras *codon 12 mutation has been reported in bladder cancers [41]. In this study, Ha-*ras*^V12 ^mutation was detected in the tumour part of the bladder cancer specimen by SNP-real-time PCR and verified by sequence analysis (Figure 1A, Gly12 -> Val 12). The Aurora-A protein overexpression was detected in the same cancer part of the bladder tissue compared to the normal part by IHC staining (Figure 1B, T vs. N). Similarly, Ki-*ras *codon 12 mutation and higher expression level of Aurora-A were only detected in the cancer part of the colon tissue (Figure 1C and 1D). Taken together, despite of the difference in transformation of NIH3T3 cells by Ki-*ras *and Ha-*ras*, overexpression of Aurora-A and Ras^V12 ^(Ki- or Ha-) mutations are simultaneously detected in various cancers including bladder and colon.

### Establishment of stable cell lines over-expressing Aurora-A and mutant Ras^V12^

**Figure 2:**
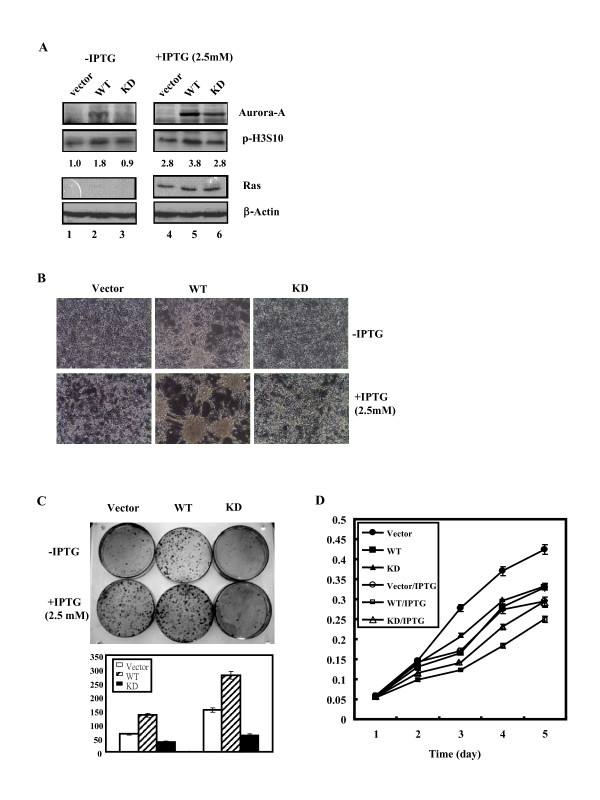
**Stable overexpression of wild-type or kinase-dead Aurora-A in WT and KD cells**. (A) Expression levels of human Aurora-A protein (wild-type: WT; kinase-inactivated: KD) and Ras in the Vector, WT and KD cells were detected by western blot analysis using anti-Aurora-A and anti-Ras specific antibodies. β-actin was used as the equal loading control. The levels of p-H3S10 protein were quantified by a densitometer. The expression level of p-H3S10 in Vector cells without IPTG induction was set as 1.0 fold. (B) Morphology of Vector, WT and KD cells with or without IPTG induction. (C) Upper panel: Cells (5 × 10^2^) were grown in 10 cm culture dishes with or without IPTG inductionfor two weeks. The foci were stained using Giemsa stain. Lower panel: Quantitative foci numbers of Vector, WT and KD cells with or without IPTG induction. (D) Growth curve of cell lines was measured daily for 5 days using MTT assay. The experiments were conducted in triplicate and repeated three times.

It is intriguing to unravel the combined effects of Aurora-A and mutant Ras^V12 ^on the morphological change and tumorigenesis of the cells. Stable cell lines were established by transfecting Vector DNA, wild-type Aurora-A or kinase-inactivated Aurora-A into 7-4 cells, which was derived from NIH/3T3 cells harboring the inducible Ha-*ras*^V12 ^oncogene [23], designated Vector, WT and KD cell line, respectively. The expression levels of Ha-*ras*^V12 ^in Vector, WT and KD cells in the presence of IPTG were much higher compared to the cells without IPTG (Figure 2A). Aurora-A can physically interact with the tail of Histone H3 (H3) and efficiently phosphorylates H3 at serine10 [45-48]. In addition, activation of ERK pathway in Ha-*ras *transformed mouse fibroblasts increases the level of p-H3S10. Consistently, our data showed the level of phosphorylated H3S10 (p-H3S10 detected by anti-p-H3S10 antibody) in WT cells (Figure 2A, lane 2, 1.8 fold) was higher than in Vector cells (Figure 2A, lane 1, 1.0 fold) and KD cells (Figure 2A, lane 3, 0.9 fold) in the absent of IPTG where Ras was not overexpressed. Our data showed that the Aurora-A overexpressed in WT cells is functional. In the presence of IPTG, where Ras^V12 ^protein was overexpressed, the level of phosphorylated H3S10 was increased both in Vector (Figure 2A, lane 4, 2.8 fold), WT (Figure 2A, lane 5, 3.8 fold) and KD (Figure 2A, lane 6, 2.8 fold) cells.

Biological activity analysis showed that WT cells over-expressing wild-type Aurora-A became rounded and formed aggregates in the presence of IPTG compared to the Vector cells and KD cells (Figure 2B). Transforming analysis showed that WT cells form more foci compared to Vector and KD cells (Figure 2C). Despite the fact that focus numbers were also increased in the other two cell lines, a further increase of focus number in WT cells was observed after IPTG induction (Figure 2C). Taken together, both Aurora-A and mutant Ras^V12 ^overexpression can induce focus formation. Further induction of focus formation was detected when these two genes were overexpressed simultaneously.

Cell proliferation analysis showed that WT cells grew slower than Vector and KD cells in the absence of IPTG. Growth rate of Vector, WT and KD cells were decreased when mutant Ras was overexpressed (Fig. 2D). The increase of cell aggregation of WT cells in the presence of IPTG was independent of cell growth rate.

### Aurora-A overexpression increases phosphorylation status of MEK/ERK and AKT as well as the activity of RalA in the Ras^V12 ^transformants

**Figure 3:**
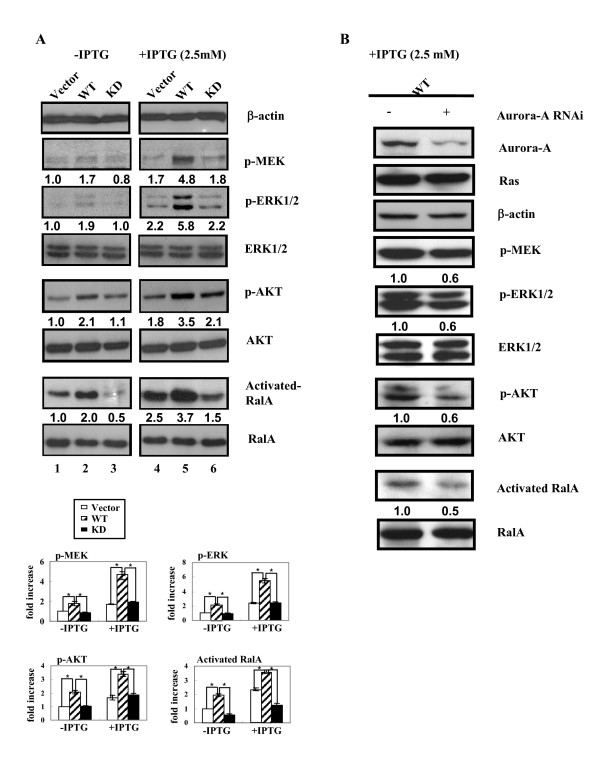
**Wild-type Aurora-A increases phosphorylation of MEK/ERK, AKT and the activity of RalA of Ras^V12 ^transformants**. (A) The phosphorylation of MEK was detected by anti-p-MEK antibody. The expression levels of AKT and p-AKT were detected using anti-AKT and anti-p-AKT specific antibodies.Ral A activity was detected by Ral pull-down assay. The expression level of each protein in three cells was shown graphically at the bottom panel. (B) After Aurora-A siRNA (5 μg) was transfected into WT cells for 6 h, IPTG was added and incubated for another 48 h. The total cell lysates (100 μg) were separated by 12% SDS-PAGE. Ral A, Ras, Aurora-A and β-actin were detected using Western blotting. The level of each protein was quantified by a densitometer. The expression level of each protein in Vector cells without IPTG induction was set as 1.0 fold. " * ": p ≦ 0.01

To clarify the effects of Aurora-A on the signaling pathways related to Ras overexpression, three downstream signaling pathways of Ras, Raf/MEK, PI3K/AKT and RalGDS/Ral A were investigated. The phosphorylation of MEK (p-MEK) was higher in WT cells (Figure 3A, lane 2, 1.7 fold) than that in Vector (Figure 3A, lane 1, 1.0 fold) and KD cells (Figure 3A, lane 3, 0.8 fold). P-MEK levels in each cell line were further increased after IPTG induction (Ras^V12 ^is over-expressed) (Figure 3A, lane 4, 5, and 6). The same phenomenon was also been observed in p-ERK1/2 (Figure 3A). These results indicated that Aurora-A may further increase Ras induced MEK/ERK phosphorylation.

The effect of Aurora-A on the PI3K/AKT pathway was evaluated by detecting phosphorylation of AKT (p-AKT). The p-AKT level was also higher in WT cells (Figure 3A, lane 2, 2.1 fold) compared to Vector and KD cells (Figure 3A, lane 1, 1.0 fold and lane 3, 1.1 fold, respectively). Upon IPTG induction, Ras^V12 ^overexpression increased the level of p-AKT in Vector and KD cells (Figure 3A, lane 4, 1.8 fold and lane 6, 2.1 fold, respectively). Co-expression of Ras^V12 ^and wild-type Aurora-A in WT cells increases the level of p-AKT (Figure 3A, lane 5, 3.5 fold) as compared to Ras^V12^overexpression alone (Figure 3A, lane 4, 1.8 fold).

The RalGDS/RalA signaling pathway was determined by detecting the activity of RalA using GST-RalBD pull-down assay. As shown in Figure 3A, Aurora-A overexpression alone activated RalA (Figure 3A, lane 2, 2.0 fold) as compared to the parental Vector cells (Figure 3A, lane 1, 1.0 fold). After IPTG induction, The RalA activity was increased by Ras^V12 ^overexpression (Figure 3A, lane 4, 2.5 fold). Co-expression of Ras^V12 ^and wild-type Aurora-A in WT cells increase the activity of RalA of Ras^V12 ^(Figure 3A, lane 5, 3.7 fold). Taken together, both Aurora-A and Ras^V12 ^increased the levels of p-MEK, pERK1/2, and p-AKT and the activation of RalA. This induction was further enhanced when Aurora-A and Ras^V12 ^were overexpressed simultaneously.

To further confirm our results, Aurora-A specific small interference RNA (siRNA) was used. As shown in Figure 3B, Aurora-A specific siRNA decreased the expression level of Aurora-A in WT cells. Accordingly, levels of p-MEK/p-ERK, p-AKT and activation of RalA were also decreased when Aurora-A siRNA was introduced into WT cells upon IPTG induction. Our results confirmed that wild-type-Aurora-A enhance Ras downstream signaling pathways including MEK/ERK, AKT and RalA.

### The MEK/ERK pathway is involved in WT cell aggregation

**Figure 4:**
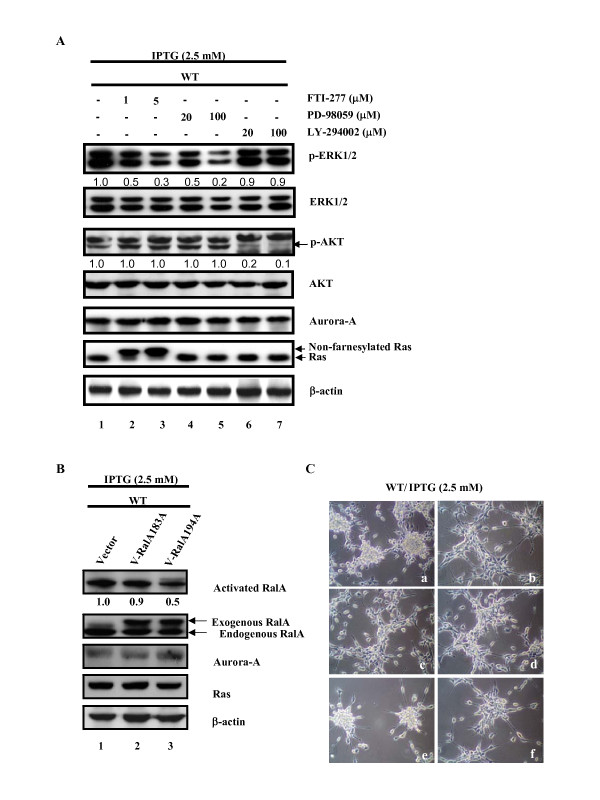
**The MEK/ERK pathway is involved in the aggregation of WT cells**. (A) Cells(5 × 10^5^) were seeded in 60 mm plates. After IPTG induction for 24 h, the signaling pathway inhibitors were added and incubated for another 24 h. The p-ERK, ERK, p-AKT, AKT, Ras, Aurora-A and β-actin were detected by western blotting. The level of each protein was quantified by a densitometer. The expression level of each protein in Vector cells without IPTG induction was set as 1.0 fold. (B) Cells(5 × 10^5^) were seeded in 60 mm plates.After transfection of WT cells for 6 h with pHA-Vector (5 μg), pHA-RalA183A (5 μg) or pHA-RalA194A (5 μg), IPTG was added and incubated for another 48 h. The total cell lysates (500 μg) were used to detect the activity of Ral A by Ral A pull-down assay. (C) Cells (5 × 10^5^) were seeded in 60 mm plates. After IPTG induction for 24 h, the inhibitors were added and incubated for another 24 h. For Aurora-A RNAi and RalAS194A, after transfection of cells for 6 h with Aurora-A siRNA (5 μg) or pHA-RalA194A (5 μg), IPTG was added and incubated for another 48 h. a.WT cells treated with IPTG. b.WT cells treated with IPTG/Aurora-A siRNA c.WT cells treated with IPTG/FTI-277 d.WT cells treated with IPTG/PD98059 e.WT cells treated with IPTG/LY294002 f.WT cells treated with IPTG/pHA-RalA194A. FTI-277, a farnesylation inhibitor of Ras; PD98059, an inhibitor of MEK kinase; LY294002, an inhibitor of PI3K kinase.

The involvement of MEK/ERK, PI3K/AKT and RalGDS/RalA signaling pathways in Aurora-A-related cell aggregation (Fig. 2B, WT + IPTG) was clarified by treatment of the cells with the following inhibitors: FTI-277, a farnesylation inhibitor of Ras; PD-98059, the inhibitor of MEK kinase and LY-294002, the inhibitor of PI3K kinase and RalASa94A, a mutant of Ral. FTI-277 restrains Ras protein as a non-farnesylated form and inhibits p-ERK1/2 expression dose-dependently but had no effect on p-AKT (Figure 4A, lanes 2 and 3). PD-98059 decreased the phosphorylation of ERK1/2 (p-ERK1/2) but had no effect on other signaling pathways (Figure 4A, lanes 4 and 5). LY-294002 reduced the phosphorylation of AKT (p-AKT) but had no effect on another signaling pathway (Figure 4A, lanes 6 and 7). In summary, in WT cells Aurora-A increases the expression of p-ERK1/2 in a Ras dependent manner. However, FTI-277 does not reduce the p-AKT in WT cells co-expressing Ras^V12 ^and wild-type Aurora-A. Wild-type Aurora-A activates RalA (Figure 3A and 3B) and phosphorylates RalA at serine194 to promote cellular transformation and migration [38]. To reveal the role of RalA phosphorylation at ser194 in Aurora-A induced RalA activation in WT cells, the mutants RalAS183A or RalAS194A were transiently transfected into WT cells and the RalA activity was evaluated. Consistent with a previous report [38], only RalAS194A could reduce the Ral A activity (Figure 4B, lane 3).

To determine which signaling pathway is involved in the aggregation of WT cells during Ras^V12 ^overexpression, we first demonstrated that Aurora-A induced cell aggregation was blocked by Aurora-A specific small interfering RNA (Figure 4C). The WT cells were treated with FTI-277, PD-98059 or LY-294002 for 24 h and cell aggregation was observed. Both FTI-277 and PD98059 reversed the aggregation of WT cells, whereas LY-294002 showed no effect on cell aggregation (Figure 4C). Because mutant RalAS194A was unable to block cell aggregation, its role in Aurora-A induced cell aggregation was excluded (Figure 4C). Taken together, the Ras/MEK/ERK signaling pathway but not the PI3K/AKT or RalGDS/RalA pathway is responsible for Aurora-A induced cell aggregation.

## Discussion

Overexpression of an oncogene such as *ras *may cause senescence of transformed cells, and this event can be reversed by overexpression of a second oncogene such as c-*myc*, and Twst1/2 [49,50]. Aurora-A can promote the cell transformation of Ha-*ras *transformed BALB/c 3T3 A31-1-1 cells [39]. The nuclear EGFR induced by EGF associates with Stat5 to bind and increase Aurora-A gene expression, which ultimately leads to chromosome instability and tumorigenesis [51]. We previously reported that oncogenic Ras-induced morphological changes (from spindle-shaped to round) occur through the MEK/ERK signaling pathway to down-regulate Stat3 at a posttranslational level in NIH3T3 cells. Microtubule disruption is involved in the morphologic changes, which can be reversed by overexpression of Stat3 [52]. In this study, we determine that overexpression of wild-type-Aurora-A can enhance Ha-*ras*^V12 ^transformant aggregation through the MEK/ERK signaling pathway.

The effector domain mutant of oncogenic Ras, Ras^V12S35^, which specifically activates the Raf/MEK/ERK pathway in transformed NIH3T3 cells, can induce subcutaneous tumor formation and lung metastases. In these Ras^V12S35^-transformed NIH 3T3 cells, high levels of activated ERK1/2 were detected. By contrast, the cells derived from the other effector domain mutants, Ras^V12G37 ^(PI3K) or Ras^V12C40 ^(RalGDS), did not show changes at the level of ERK1/2 activation and tumor metastasis [53]. The increase of ERK1/2 activation could lead to enhanced expression of many proteolysis enzymes such as the matrix metalloprotease (MMP) family genes which can degrade extracellular matrix, leading to increased cell invasiveness [54,55]. Furthermore, Aurora-A-regulated epithelial-mesenchymal transition and invasion are mediated by mitogen-activated protein kinase (MAPK) phosphorylation [58]. Our current and previous studies reveal that Ras^V12 ^mutation and Aurora-A overexpression can be detected simultaneously in human bladder and colon cancers (Figure 1). Co-expression of wild-type Aurora-A and mutant Ras enhances the signaling of the MEK/ERK, AKT and RalA activity (Figure 3). I

The activation of ERK1/2 requires phosphorylation of the conserved tyrosine and threonine residues by dual specific MAPK kinases (MEK), which are activated by the serine/threonine kinase Raf through phosphorylation. Scaffolding proteins such as MEK partner (MPI) or kinase suppressor of Ras (KSR) enhance the MEK/ERK signaling pathway in response to different stimuli [36,56-66]. The KSR/MEK complex is recruited to the membrane following dephosphorylation by phosphatase 2A (PP2A) at the Ser392 residue leading to release 14-3-3 from KSR and then exposes the C1 domain, which is required for the membrane localization of KSR, as well as the FxFP MAPK binding site. At the membrane, Raf-1 is activated and KSR provides a platform for the phosphorylation/activation of associated MEK and ERK [62,65]. Other proteins might help recruit activated Raf, triggering MEK phosphorylation. PP2A also interacts with Aurora-A [67]. Whether the PP2A may regulate Aurora-A and KSR complex to affect the MEK/ERK signaling pathway is valuable to explore. In addition, Aurora-A interacts with the other tumor suppressor RASSF1A. Aurora-A phosphorylates RASSF1A at Threonine202 and/or Serine203. Knockdown of RASSF1A reduces Aurora-A activation; however, the recombinant RASSF1A can not activate recombinant Aurora-A *in vitro *suggesting that RASSF1A may function as a scaffold for Aurora-A activation [68,69]. The possibility of the interaction between Aurora-A and KSR or RASSF1A requires more investigation and the involvement of other unidentified factor(s) in ERK1/2 activation induced by Aurora-A in R*as*^V12 ^transformants can not be excluded.

PI3K/AKT is a down stream signaling pathway of Ras. In Figure 3A, Ras^V12 ^or Wild-type Aurora-A alone increases the p-AKT level (Figure 3A, lane 2 and 4) and further increase p-AKT while both of the genes were overexpressed (Figure 3A, lane 5). However, upon FTI-277 treatment, the p-AKT level was not reduced in WT cell when RasV12 was overexpressed (Figure 4A, lane 2 and 3). Above results suggest that Ras^V12 ^and wild-type Aurora-A may share a redundant pathway to increase p-AKT expression level. Nonetheless, the underlying mechanism is unclear.

Overexpression of Aurora-A induces cell motility of MDCK cells, mediated by RalA activation through phosphorylation of the serine 194 residue of RalA [38]. In the present study, we demonstrated that overexpression of either Aurora-A or mutant Ras stimulates RalA activation and maximal RalA activation is observed when both of the oncogenes are overexpressed (Fig. 3A, lane 5). However, we found that the RalAS194A mutant could not block cell aggregation induced by Aurora-A in the Ha-*ras*^V12 ^transformants indicating that different signaling pathways may be transduced to control motility and aggregation of the different cells.

In summary, our data demonstrate that aberrant Aurora-A expression plus *ras *mutation may occur simultaneously in various cancers, and the increase of MEK/ERK activation triggered by over-expression of the two oncogenes induces cell aggregation. We speculate that this event may play a pivotal role in Ras or Aurora-A related tumor progression.

## Conclusions

Taken together, both Aurora-A and Ras^V12 ^mutant can activate the MEK/ERK1/2 signaling pathway. Our study reveals that additional activation of ERK1/2 may induce cell aggregation and increase cell focus formation when both oncogenes are overexpressed together. The results suggest that increased risk of tumor progression is possible through increase of ERK1/2 phosphorylation by diverse oncogenes.

## Competing interests

The authors declare that they have no competing interests.

## Authors' contributions

YST participated in conceptualization, carried out this study, and drafted the manuscript; JCL participated in the collection of cancer tissues; CYFH provided the Aurora-A wild and kinase dead plasmids as well as RalAS194A and RalAS183A plasmids. HSL conceived of the study, and participated in its design and coordination. All authors read and approved the final manuscript.

## Pre-publication history

The pre-publication history for this paper can be accessed here:

http://www.biomedcentral.com/1471-2407/9/435/prepub
